# A Link between Prenatal Stage of Life during the Great Chinese Famine and Subsequent Depressive Symptoms among Middle-Aged and Older Adults

**DOI:** 10.3390/nu15214600

**Published:** 2023-10-29

**Authors:** Yushan Du, Yanan Luo, Lirong Nie, Ziyang Ren, Jinfang Sun, Jufen Liu

**Affiliations:** 1 Institute of Reproductive and Child Health/National Health Commission Key Laboratory of Reproductive Health, Peking University, Beijing 100191, China; duyushan_kally@163.com (Y.D.); nielirong99@163.com (L.N.); ziyang_ren@bjmu.edu.cn (Z.R.); 2 Department of Epidemiology and Biostatistics, School of Public Health, Peking University, Beijing 100191, China; 3 Department of Global Health, School of Public Health, Peking University, Beijing 100191, China; luoyanan@bjmu.edu.cn; 4 Office of Epidemiology, Chinese Center for Disease Control and Prevention, Beijing 102206, China

**Keywords:** prenatal malnutrition, Chinese Great Famine, depressive symptoms, natural experiment

## Abstract

Prenatal malnutrition may increase the risk of depressive symptoms in adulthood. This study investigated the association between prenatal exposure to malnutrition with risk of depressive symptoms in middle-aged and older adults using the Chinese Great Famine of 1959–1961 as a natural experiment. Data were obtained from the China Health and Retirement Longitudinal Study baseline survey (2011). A total of 5391 individuals born from 1956 to 1965 were included in the study. Depressive symptoms were ascertained via the Center for Epidemiological Studies Depression Scale short form. Famine severity was measured using the cohort size shrinkage index. Difference-in-differences models were used to explore the association between prenatal famine exposure and later-life depressive symptoms. Compared with the post-famine cohort (1963–1965), famine cohorts (1959–1962) were 4.74 times (95% CI = 1.28–8.20) as likely to develop depressive symptoms. The stratified analysis found that prenatal exposure to famine was associated with depressive symptoms in rural residents but not those living in urban areas. In rural females, prenatal malnutrition was associated with a higher risk of depressive symptoms. However, there was no significant association between prenatal malnutrition and depressive symptoms in rural males. Our results indicated that prenatal malnutrition may contribute to a higher risk for depressive symptoms in later life among female rural residents.

## 1. Introduction

Depressive disorders are one of the most common mental health disorders. It has been estimated that depressive disorders affect 264.4 million people globally and are one of the leading causes of both Years Lived with Disability and Disability Adjusted Life Year [1]. In China, 24.1% of middle-aged and older adults suffer from depressive symptoms [2], and depressive symptoms contribute to a heavy health burden with a high economic cost, accounting for an estimated 7.8% of the total personal expected medical cost [3]. Older age has been identified to be an important risk factor for the aggravation of the depressive disorder course [4]. Therefore, with the rapidly aging population in China, early detection and prevention of depressive symptoms is necessary.

From a life course perspective, early life experiences can have a long–term impact on health throughout an entire lifetime. Infant health and development are crucial and can have a great impact on health in adulthood [5]. Fetal life is the beginning of the “first 1000 days” of the life course, which is essential for later health trajectories [6]. Malnutrition during pregnancy has a direct effect on infant mortality; furthermore, it can lead to profound consequences and adversely affect the lifelong health of the offspring [7]. Mounting evidence suggests that early life food deprivation has a negative lasting effect on mental health throughout the life course [8,9]. Additionally, a higher risk of depressive symptoms late in life has been found to be related to malnutrition in early life [10]. Researchers have increasingly used famine exposure as a natural experiment to examine the long-term health effects of prenatal malnutrition [11]. While the findings about the association between famine exposure and depressive symptoms remain controversial [12,13,14], this controversy may be attributed to varied cultures and histories and different severities of famine exposure. Nevertheless, these findings imply that malnutrition exposure in early life may play a long-term role in depressive symptoms to some extent.

The Chinese Great Famine, which occurred between 1959 and 1961, provides a unique opportunity for us to explore the relationship between prenatal malnutrition and depressive symptoms [15,16]. China’s 1959–1961 famine is regarded as the largest famine in human history [17]. Both the severe drought of 1960–1961 and the policy failures of the Great Leap Forward Campaign were considered to be the main contributors to this catastrophic event [9]. The Chinese famine of 1959–1961 caused an estimated 15–30 million excess deaths, and about 30 million births did not take place because people made decisions to delay or forgo childbearing [18]. In China, cohorts who experienced prenatal exposure to famine represent groups with cumulative disadvantages and vulnerabilities throughout the life cycle and face more health challenges when confronted with adverse risks [19,20]. Only a few studies focused on the association between early-life famine exposure and later-life depressive symptoms. Furthermore, the existing studies of famine exposure and depressive symptoms did not take into account the variation in famine severity across different regions or the use of self-reported measurement to assess severity, which may lead to recall and estimated bias [21,22]. Taking the Chinese Great Famine as a quasi-natural experiment, this study examined the association between prenatal malnutrition and the risk of depressive symptoms in later life based on difference-in-differences (DID) models, which provided a life course perspective for depressive symptoms prevention.

## 2. Materials and Methods

### 2.1. Study Participants

We used data from the baseline survey of the China Health and Retirement Longitudinal Study (CHARLS). CHARLS is a nationally representative longitudinal survey of adults aged 45 years and older and their spouses. The 2011 baseline survey for the study was conducted between June 2011 and March 2012. The survey used a multistage probability sampling design and sampled 17,708 individuals, and all of them signed informed consent. More details about this survey are described elsewhere [23]. The CHARLS programme protocol was approved by the ethical committee of Peking University (approval code: IRB00001052-11015).

For purposes of this analysis, we selected participants with prenatal exposure to the Chinese Great Famine. “Prenatal exposure” was defined as “maternal exposure to famine during the approximately 300 days between the periconceptional and perinatal period” [24]. In this study, 6337 participants born between 1956 and 1965 aged 46–55 at the time of the survey were selected. We defined the 1959–1962 birth cohorts as the famine cohort (aged 49–52), the 1956–1958 birth cohorts as the pre-famine cohort (aged 53–55), and the 1963–1965 birth cohorts as the post-famine cohort (aged 46–48).

To minimize the migration impact on our estimation, 286 participants with migration experience before 16 years old were excluded. In this study, 17 participants with missing information concerning residence, sex, marital status and education and 643 participants with missing information on CES-D 10 scores were excluded. Finally, this study included 5391 participants (see Figure S1). We compared the distribution of three birth cohorts (pre-famine, mid-famine, and post-famine) among the total participants and those included in our study, and there were no statistically significant differences in the proportions of these birth cohorts between the two groups (*p* > 0.05).

### 2.2. Measures

#### 2.2.1. Depressive Symptoms

The Center for Epidemiological Studies Depression Scale (CES-D) short form was used to measure depressive symptoms in the baseline survey of the CHARLS. The CES-D short form (CES-D 10) is a structured self-report scale with 10 items. For each item, participants reported the frequency of occurrence for the item during the past week (≤1 day per week, 1–2 days per week, 3–4 days per week, 5–7 days per week). The sum score of CES-D 10 ranges from 0 to 30, with higher scores indicating higher levels of depressive symptoms [25]. The CES-D 10 was proven to be a useful mental health measure for old adults in China (Cronbach’s alpha = 0.78–0.79) [26] and showed adequate reliability and validity among community-dwelling older participants in CHARLS [27].

#### 2.2.2. Famine Severity

The cohort size shrinkage index (CSSI) was used to measure famine severity at the province level [9,28,29,30,31]. The assumption was that the smaller size of the cohorts born during the famine years compared to the surrounding cohorts is caused by reduced fertility and/or increased mortality, and the smaller size indicates the greater severity of the famine. The average cohort size of those born during the famine (1959–1961) was defined as N_fam_, and the average cohort size of those born three years after the famine (1962–1964) was defined as N_nonfam_. The sizes of the cohorts were obtained from the China Statistical Yearbook, and CSSI was calculated as (N_nonfam_ − N_fam_)/N_nonfam_. The validity and reliability of CSSI have been shown to be reasonably good [9,28]. Due to the administrative region division of China during 1956–1965, Tianjin, Hainan and Chongqing were included in Hebei, Guangdong and Sichuan, respectively. Tibet was excluded due to a lack of population data during the famine period. Therefore, 27 provinces’ CSSI values were included in our analysis. These CSSI values varied between 0.06 and 0.62, with an average value of 0.36. CSSI values of 27 provinces were calculated by Zheng et al. [29].

#### 2.2.3. Covariates

Covariates included sex, residence, marital status and education. Residence was divided into two groups: rural and urban. Marital status was categorized as follows: living with spouse (including ‘married and living with spouse’) and living without spouse (including ‘married but not living with spouse temporarily’, ‘separated’, ‘divorced’, ‘widowed’, and ‘never married’). Education was categorized as follows: primary school or below (i.e., with educational attainment of ≤6 years) and junior high school or above (i.e., with educational attainment of >6 years).

### 2.3. Statistical Analysis

The description of CES-D 10 scores is presented as medians with interquartile ranges (IQRs). IQR was used to measure the difference between the first quartile (25th percentile) and third quartile (75th percentile) and the difference between the first quartile and third quartile of CES-D 10 scores. Additionally, the description of sex, residence, marital status and education are presented as frequencies and percentages. The Kruskal–Wallis test was used to compare continuous variables, and the chi-square test was used to compare categorical variables. Dunn’s tests with Bonferroni correction were performed for multiple pairwise comparisons.

The association between famine exposure and the risk of depressive symptoms was estimated via the DID model, which examined the regional variation in famine exposure across birth cohorts. The DID model can permit intrinsic cohort differences and define famine exposure at the local level, which makes the estimation more robust [32,33]. Regression models with DID estimators were fitted as follows:

(1) $${Y_{ijk}=\beta }_{0}+\delta_{j}{CSSI}_{j}+\varphi_{k}{Cohort}_{k}+\beta_{jk}({CSSI}_{j}\times {Cohort}_{k})+{VX}_{ijk}+\varepsilon_{ijk}$$

where $Y_{ijk}$ is the CES-D 10 scores for individual i born in province j, and from birth cohort k, with k taking values from 1 to 3. k = 1 refers to pre-famine cohort born in 1956–1958, k = 2 refers to famine cohort born in 1959–1962, and k = 3 refers to post-famine cohort born in 1963–1965 (i.e., reference group). $\beta_{0}$ represents the intercept term of the model. ${CSSI}_{j}$ represents the famine severity of province j, $\varphi_{k}$ represents the cohort fixed effect, ${VX}_{ijk}$ denotes a vector of control variables, and $\varepsilon_{ijk}$ is random errors.

The interaction coefficient between the CSSI and the birth cohort dummy variables, namely, $\beta_{jk}$, evaluates the association between prenatal famine exposure and the risk of higher CES-D 10 scores in the DID model.

Additionally, to examine the validity and robustness of DID estimates, we used the new control cohort (born in 1949–1953) and the placebo-treated cohort (born in 1954–1958) to perform a placebo test. An interaction coefficient between the CSSI and the birth cohort dummy variables with no statistical significance suggests that the association estimated via DID is robust.

The software Stata version 15 for Windows (Stata Corp, College Station, TX, USA) was used to perform statistical analyses, and a two-sided *p* < 0.05 was considered significant.

## 3. Results

### 3.1. Basic Characteristics

A total of 5391 participants were included in the study. A total of 30.1% of participants were born before the famine, 33.6% were born during the famine, and 36.3% were born after the famine. In total, 54.3% of participants were females. Most of the participants were born and lived in rural areas and were living with spouses. A total of 51.1% of the participants had junior high school and above education. Of 5391 middle-aged and older adults, the median CES-D 10 score was 6.0 (8.0).

There were statistically significant differences among the three birth cohorts (pre-famine, famine, and post-famine) in sex, education and CES-D 10 scores (*p* < 0.05). No statistically significant differences were observed among the three groups in residence and marital status (*p* > 0.05). There were significant differences in CES-D 10 scores between pre-famine and post-famine cohorts and between famine and post-famine cohorts (*p* < 0.05). Additionally, there were no significant differences in CES-D 10 scores between the pre-famine and famine cohorts (*p* > 0.05). More details can be found in Table 1.

### 3.2. Comparisons of CES-D 10 Scores

There were statistically significant differences among the three cohorts (pre-famine, famine, and post-famine) in CES-D 10 scores stratified in terms of sex, residence, marital status and education (*p* < 0.05). Participants with higher CES-D 10 scores were more likely to be females, rural residents, individuals living without spouses, and individuals with lower levels of educational attainment (Table 2).

### 3.3. Famine Exposure and Risk of Depressive Symptoms

The DID model estimated the association between famine exposure and CES-D 10 scores, and the interaction coefficients between CSSI (famine exposure) and the birth cohorts are shown in Table 3. After controlling marital status and education, the coefficient of the CSSI × Famine cohort remained statistically significant, indicating that the famine significantly increased the CES-D 10 scores. In our subgroup analysis, the association between prenatal exposure to famine and depressive symptoms was only significant among rural residents. We also conducted the standard placebo test to probe the robustness of our main results. We defined the 1954–1958 birth cohorts as the placebo-treated cohorts, and the 1950–1953 birth cohorts as the control cohorts. The standard placebo test shows no signs of statistical significance (Table S3).

Table 4 and Table 5 show the associations of famine exposure with CES-D 10 scores in rural and urban participants. This relationship between prenatal famine exposure and higher CES-D 10 scores was only significant in rural females.

## 4. Discussion

Taking the Chinese Great Famine of 1959–1961 as a natural experiment, this study investigated the association between prenatal exposure to malnutrition and the risk of depressive symptoms among Chinese middle-aged and older adults by using a nationally representative survey. The prevalence of depressive symptoms in our study (23.0%) is generally consistent with another nationally representative study (24.1%) [2]. Our findings indicated that prenatal famine exposure was associated with an increased prevalence of depressive symptoms in later life. The sex- and region-specific association between prenatal exposure to famine and depressive symptoms was observed: association between prenatal famine exposure and depressive symptoms was found to be significant only in rural residents, not in urban counterparts, with a higher risk of depressive symptoms noted in rural females but not in rural males. A higher risk of depressive symptoms was only in rural females but not in rural males.

To the best of our knowledge, only three studies investigated the association of the famine experience in early life with depressive symptoms in later life in the context of China [21,22,34], which found similar results to our findings. However, these studies have some limitations. For example, one of them was unconsidered for the famine severity [22]; self-report questions to measure famine severity in the other study may lead to recall bias [21]; another study incorrectly identified the perinatal famine exposure period, overlooking the 300 days of prenatal exposure to the year of the famine ended, which may lead to the loss of vital information and bias [34]. Our study used CSSI to measure the severity of the Chinese Great Famine, and we used the DID model to better control unobserved differences among birth cohorts caused by non-famine factors. These improvements will make our estimates more precise and robust and can, to some extent, fill the gaps in existing evidence.

Our findings suggest an association between prenatal exposure to famine and the risk of depressive symptoms among middle-aged and older adults in China. Two possible mechanisms may arise to explain this association: First, maternal malnutrition may affect the growth and structure of brain components such as the amygdala, prefrontal cortex, hypothalamus, and autonomic nervous system [35]. A meta-analysis has demonstrated structural and functional abnormalities of the amygdala in patients with major depression [36]. The amygdala is involved in emotion processing; therefore, abnormal growth of the amygdala in early life may increase the risk of emotional disorders in later life. Second, maternal malnutrition can lead to epigenetic changes in the fetal brain [35]. Recent evidence has shown the involvement of epigenetic processes in psychiatric disorders. Epigenetic regulatory mechanisms, such as DNA methylation and histone modification, may influence depression by modulating genes involved in its pathophysiology [37].

Specifically, we found an area-specific association between prenatal exposure to the Chinese famine and depressive symptoms, and the higher risk of later-life depressive symptoms was only in rural residents, not in urban residents. Severer famine exposure in rural areas may result in area differences in our findings. Previous evidence has demonstrated that famine exposure was much greater in rural residents than in urban residents [38]. The possible reasons may be explained as follows: First, rural per capita grain output decreased dramatically during the Great Chinese Famine [17]. Second, due to the state monopoly for purchase and marketing, the government purchased the surplus and a portion of the grain rations from farmers to ensure a basic grain supply for urban residents [39]. According to previous evidence, the infant mortality rate in rural areas was more than twice as high as that in urban areas during the famine [40]. Under these circumstances, residents in rural areas may be at a higher risk of experiencing social and material disadvantages, which is associated with a higher risk of depressive symptoms later in life.

Additionally, this study suggested a sex difference in the association between prenatal exposure to famine and depressive symptoms. For rural females, prenatal famine exposure was associated with a higher risk of later-life depressive symptoms, while this association was non-significant among rural males. Some possible reasons for this sex difference were interpreted as follows: First, disparities of resources resulting from gender inequity may contribute to sex differences in the risk of depressive symptoms among rural famine birth cohorts. Because of son preference, parents in traditional Chinese families prefer to allocate more resources, such as education opportunities, to sons than to daughters when faced with tightened resource constraints [10,41]. In our study, only 38.4% of rural females in the famine cohort had junior high school and above education, compared to 67.6% of rural males (Table S1). According to the hypothesis from resource substitution theory, the health effects of education may be stronger among those with fewer alternative resources [42]. Moreover, higher educational attainment may serve as a protective factor for later-life depressive symptoms [43]. Therefore, because of the educational attainment inequalities, rural female survivors with prenatal famine exposure may fail to get health benefits from education and have a higher risk for the development of depressive symptoms. Second, male fetuses tend to be more vulnerable to circumstantial changes (e.g., famine) compared to their female counterparts [44]. Survivorship bias may contribute to the better later-life health status of male survivors with prenatal famine exposure compared to their female counterparts [41]. Evidence from our supplementary data analysis also supports this speculation: In the rural famine cohort, female, both overall and in all subgroups, had lower CES-D 10 scores compared to their male counterparts (Table S2).

Our study had several limitations. First, this study could not exclude the confounding age effect from the cohort effect. Although our analysis strategy tried the best to minimize the age differences among cohorts, and there was no significant difference in CES-D 10 scores when comparing pre-famine and post-famine cohorts with the famine cohort, caution is still necessary when interpreting our results. Second, migration is a potential concern that may affect our estimators. However, this concern may be alleviated by the passport registration system in China, namely ‘hukou’, which strictly regulates migration [18]. Third, our estimates of the association between prenatal famine exposure and the risk of depressive symptoms might be underestimated due to the selective mortality effects. The surviving famine cohorts are likely to consist of individuals with better health endowments who are resilient to adverse environments. Fourth, we were unable to control prenatal stress exposure and other factors during famine, which may affect the brain development of offspring, even though starvation and malnutrition are the main and direct consequences of famine. In addition, residual confounding due to some unmeasured covariates may have remained. For example, the onset age of depressive symptoms was not estimated due to the lack of this variable in the questionnaire. Additional factors which may have effects on the health impact of famine should be considered in future studies. Furthermore, the relatively small sample size of subgroups resulted in wider confidence intervals in our results and limited our ability to detect the association between prenatal famine exposure and depressive symptoms among urban participants. These findings should be further confirmed in future studies with a larger number of participants.

## 5. Conclusions

In conclusion, our study suggests that prenatal exposure to the Chinese Great Famine may be associated with an increased risk of depressive symptoms among middle-aged and older female rural residents. Our findings emphasized the importance of preventing prenatal malnutrition to minimize potential long-term adverse health outcomes across the life course. For better health throughout the life course, the improvement of maternal nutrition should be given more attention by implementing nutritional interventions (e.g., preconception counseling, maternal diet quality assessment, and perinatal micronutrient supplementation).

## Figures and Tables

**Table 1:** Characteristics of participants, CHARLS, 2011, *N* (%).

Characteristics	All Participants (n = 5391)	Birth Cohorts			
		Pre-Famine (n = 1624)	Famine (n = 1812)	Post-Famine (n = 1955)	*p*
Sex					
Male	2264 (45.7)	801 (49.3)	815 (45.0)	848 (43.4)	<0.001 ^ab^
Female	2927 (54.3)	823 (50.7)	997 (55.0)	1107 (56.6)	
Residence					
Rural	4782 (88.7)	1456 (89.7)	1588 (87.6)	1738 (88.9)	0.166 ^d^
Urban	609 (11.3)	168 (10.3)	224 (12.4)	217 (11.1)	
Marital status					
Living with spouse	4758 (88.3)	1432 (88.2)	1595 (88.0)	1731 (88.5)	0.879 ^d^
Living without spouse	633 (11.7)	192 (11.8)	217 (12.0)	224 (11.5)	
Education					
Primary school and below	2639 (49.0)	937 (57.7)	806 (44.5)	896 (45.8)	<0.001 ^ab^
Junior high school and above	2752 (51.0)	687 (42.3)	1006 (55.5)	1059 (54.2)	
CES-D 10 scores, median (IQR)	6.0 (8.0)	6.0 (8.5)	6.0 (8.0)	6.0 (7.0)	0.012 ^bc^

Note: IQR, interquartile range; ^a^ difference between the pre-famine and famine cohorts (*p* < 0.05); ^b^ difference between the pre-famine and post-famine cohorts (*p* < 0.05); ^c^ difference between the famine and post-famine cohorts (*p* < 0.05); ^d^ no significant difference among the three cohorts (*p* > 0.05).

**Table 2:** Stratified distributions of CES-D 10 scores among participants, Median (IQR).

	Pre-Famine (n = 1624)	Famine (n = 1812)	Post-Famine (n = 1955)
Sex			
Male	6.0 (7.0)	5.0 (8.0)	5.0 (7.0)
Female	8.0 (10.0)	7.0 (8.0)	7.0 (8.0)
*p*	<0.001	<0.001	<0.001
Residence			
Rural	7.0 (9.0)	7.0 (9.0)	6.0 (8.0)
Urban	5.0 (7.0)	4.0 (5.0)	4.0 (6.0)
*p*	<0.001	<0.001	<0.001
Marital status			
Living with spouse	6.0 (8.0)	6.0 (8.0)	6.0 (7.0)
Living without spouse	10.0 (11.0)	9.0 (10.0)	8.0 (9.0)
*p*	<0.001	<0.001	<0.001
Education			
Primary school and below	8.0 (9.0)	8.0 (9.0)	7.0 (8.0)
Junior high school and above	5.0 (8.0)	5.0 (8.0)	5.0 (7.0)
*p*	<0.001	<0.001	<0.001

Note: IQR, interquartile range.

**Table 3:** Associations of exposure to the Chinese Great Famine with CES-D 10 scores among participants, CHARLS, 2011 (N = 5391).

	Unadjusted β (95% CI)	*p*	Adjusted β (95% CI)	*p*
Overall participants				
Post-famine cohort (1963–1965)	Reference		Reference	
Famine cohort (1959–1962)	5.54 (1.97, 9.10)	0.002	4.74 (1.28, 8.20) ^a^	0.007
Pre-famine cohort (1956–1958)	1.21 (−2.59, 5.01)	0.533	1.49 (−2.20, 5.18) ^a^	0.429
Rural				
Post-famine cohort (1963–1965)	Reference		Reference	
Famine cohort (1959–1962)	5.09 (1.10, 9.07)	0.494	4.51 (0.62, 8.39) ^b^	0.023
Pre-famine cohort (1956–1958)	1.47 (−2.75, 5.70)	0.012	1.13 (−2.99, 5.26) ^b^	0.590
Urban				
Post-famine cohort (1963–1965)	Reference		Reference	
Famine cohort (1959–1962)	4.66 (−3.31, 12.64)	0.251	4.70 (−3.09, 12.50) ^b^	0.237
Pre-famine cohort (1956–1958)	0.87 (−7.40, 9.13)	0.837	3.80 (−4.37, 11.96) ^b^	0.362

Note: CI: confidence interval; ^a^ adjusted for residence, sex, marital status, education; ^b^ adjusted for sex, marital status, education.

**Table 4:** Associations of exposure to the Chinese Great Famine with CES-D 10 scores among rural birth participants, CHARLS, 2011 (N = 4782).

	Unadjusted β (95% CI)	*p*	Adjusted ^a^ β (95% CI)	*p*
Male				
Post-famine cohort (1963–1965)	Reference		Reference	
Famine cohort (1959–1962)	2.69 (−2.84, 8.22)	0.340	1.85 (−3.57, 7.27)	0.504
Pre-famine cohort (1956–1958)	4.71 (−1.05, 10.48)	0.109	3.22 (−2.44, 8.88)	0.264
Female				
Post-famine cohort (1963–1965)	Reference		Reference	
Famine cohort (1959–1962)	6.68 (1.14, 12.23)	0.018	6.58 (1.08, 12.08)	0.019
Pre-famine cohort (1956–1958)	−0.74 (−6.72, 5.23)	0.807	−0.79 (−6.72, 5.14)	0.794

Note: CI: confidence interval; ^a^ adjusted for marital status, education.

**Table 5:** Associations of exposure to the Chinese Great Famine with CES-D 10 scores among urban birth participants, CHARLS, 2011 (N = 609).

	Unadjusted β (95% CI)	*p*	Adjusted ^a^ β (95% CI)	*p*
Male				
Post-famine cohort (1963–1965)	Reference		Reference	
Famine cohort (1959–1962)	5.06 (−7.10, 17.23)	0.413	6.42 (−5.41, 18.25)	0.286
Pre-famine cohort (1956–1958)	2.80 (−10.01, 15.61)	0.667	8.60 (−4.13, 21.33)	0.185
Female				
Post-famine cohort (1963–1965)	Reference		Reference	
Famine cohort (1959–1962)	4.32 (−6.34, 14.99)	0.426	4.20 (−6.22, 14.63)	0.428
Pre-famine cohort (1956–1958)	−0.99 (−12.04, 10.06)	0.846	0.53 (−10.28, 11.35)	0.923

Note: CI: confidence interval; ^a^ adjusted for marital status, education.

## Data Availability

Data are available in a public, open-access repository.

## Supplementary Materials

The following supporting information can be downloaded at: https://www.mdpi.com/article/10.3390/nu15214600/s1, Figure S1: Flow chart of study participants; Table S1: Characteristics of participants, CHARLS, 2011, N (%); Table S2: Stratified distributions of CES-D 10 scores among participants, CHARLS, 2011, median (IQR); Table S3: The placebo test of difference-in-differences estimates of prenatal famine exposure and depressive symptoms.

## References

[1] GBD 2017 Disease and Injury Incidence and Prevalence Collaborators (2018). Global, regional, and national incidence, prevalence, and years lived with disability for 354 diseases and injuries for 195 countries and territories, 1990–2017: A systematic analysis for the Global Burden of Disease Study 2017. Lancet.

[2] Fan X., Guo X., Ren Z., Li X., He M., Shi H., Zha S., Qiao S., Zhao H., Li Y. (2021). The prevalence of depressive symptoms and associated factors in middle-aged and elderly Chinese people. J. Affect. Disord..

[3] Hsieh C.R., Qin X. (2018). Depression hurts, depression costs: The medical spending attributable to depression and depressive symptoms in China. Health Econ..

[4] Schaakxs R., Comijs H.C., Lamers F., Kok R.M., Beekman A.T.F., Penninx B. (2018). Associations between age and the course of major depressive disorder: A 2-year longitudinal cohort study. Lancet. Psychiatry.

[5] Wadsworth M.E. (1997). Health inequalities in the life course perspective. Soc. Sci. Med..

[6] Aagaard-Hansen J., Norris S.A., Maindal H.T., Hanson M., Fall C. (2019). What are the public health implications of the life course perspective?. Glob. Health Action.

[7] Scott S., Duncan S.R., Duncan C.J. (1995). Infant mortality and famine: A study in historical epidemiology in northern England. J. Epidemiol. Community Health.

[8] van den Broek T., Fleischmann M. (2019). Prenatal famine exposure and mental health in later midlife. Aging Ment. Health.

[9] Huang C., Phillips M.R., Zhang Y., Zhang J., Shi Q., Song Z., Ding Z., Pang S., Martorell R. (2013). Malnutrition in early life and adult mental health: Evidence from a natural experiment. Soc. Sci. Med..

[10] Cui H., Smith J.P., Zhao Y. (2020). Early-life Deprivation and Health Outcomes in Adulthood: Evidence from Childhood Hunger Episodes of Middle-aged and Elderly Chinese. J. Dev. Econ..

[11] Lumey L.H., Stein A.D., Susser E. (2011). Prenatal famine and adult health. Annu. Rev. Public Health.

[12] Jürges H., Kopetsch T. (2021). Prenatal exposure to the German food crisis 1944–1948 and health after 65 years. Econ. Hum. Biol..

[13] Stein A.D., Pierik F.H., Verrips G.H., Susser E.S., Lumey L.H. (2009). Maternal exposure to the Dutch famine before conception and during pregnancy: Quality of life and depressive symptoms in adult offspring. Epidemiology.

[14] de Rooij S.R., Painter R.C., Phillips D.I., Räikkönen K., Schene A.H., Roseboom T.J. (2011). Self-reported depression and anxiety after prenatal famine exposure: Mediation by cardio-metabolic pathology?. J. Dev. Orig. Health Dis..

[15] Xu H., Li L., Zhang Z., Liu J. (2016). Is natural experiment a cure? Re-examining the long-term health effects of China’s 1959–1961 famine. Soc. Sci. Med..

[16] Cheng Q., Trangucci R., Nelson K.N., Fu W., Collender P.A., Head J.R., Hoover C.M., Skaff N.K., Li T., Li X. (2020). Prenatal and early-life exposure to the Great Chinese Famine increased the risk of tuberculosis in adulthood across two generations. Proc. Natl. Acad. Sci. USA.

[17] Smil V. (1999). China’s great famine: 40 years later. BMJ.

[18] Chen Y., Zhou L.A. (2007). The long-term health and economic consequences of the 1959–1961 famine in China. J. Health Econ..

[19] Dannefer D. (2020). Systemic and Reflexive: Foundations of Cumulative Dis/Advantage and Life-Course Processes. J. Gerontol. Ser. B Psychol. Sci. Soc. Sci..

[20] LI Q., Deng J., Xiao Z. (1999). Social transformation and personal development: Paradigm and methods of the life course research. Sociol. Stud..

[21] Li C., Miles T., Shen L., Shen Y., Liu T., Zhang M., Li S., Huang C. (2018). Early-life exposure to severe famine and subsequent risk of depressive symptoms in late adulthood: The China Health and Retirement Longitudinal Study. Br. J. Psychiatry J. Ment. Sci..

[22] Li Y., Zhao L., Yu D., Ding G. (2018). Exposure to the Chinese famine in early life and depression in adulthood. Psychol. Health Med..

[23] Zhao Y., Hu Y., Smith J.P., Strauss J., Yang G. (2014). Cohort profile: The China Health and Retirement Longitudinal Study (CHARLS). Int. J. Epidemiol..

[24] Susser E., St Clair D. (2013). Prenatal famine and adult mental illness: Interpreting concordant and discordant results from the Dutch and Chinese Famines. Soc. Sci. Med..

[25] Kohout F.J., Berkman L.F., Evans D.A., Cornoni-Huntley J. (1993). Two shorter forms of the CES-D (Center for Epidemiological Studies Depression) depression symptoms index. J. Aging Health.

[26] Boey K.W. (1999). Cross-validation of a short form of the CES-D in Chinese elderly. Int. J. Geriatr. Psychiatry.

[27] Chen H., Mui A.C. (2014). Factorial validity of the Center for Epidemiologic Studies Depression Scale short form in older population in China. Int. Psychogeriatr..

[28] Huang C., Li Z., Narayan K.M., Williamson D.F., Martorell R. (2010). Bigger babies born to women survivors of the 1959–1961 Chinese famine: A puzzle due to survival selection?. J. Dev. Orig. Health Dis..

[29] He P., Chen G., Guo C., Wen X., Song X., Zheng X. (2018). Long-term effect of prenatal exposure to malnutrition on risk of schizophrenia in adulthood: Evidence from the Chinese famine of 1959–1961. Eur. Psychiatry J. Assoc. Eur. Psychiatr..

[30] Huang C., Guo C., Nichols C., Chen S., Martorell R. (2014). Elevated levels of protein in urine in adulthood after exposure to the Chinese famine of 1959–61 during gestation and the early postnatal period. Int. J. Epidemiol..

[31] Huang C., Li Z., Wang M., Martorell R. (2010). Early life exposure to the 1959–1961 Chinese famine has long-term health consequences. J. Nutr..

[32] Li C., Lumey L.H. (2017). Exposure to the Chinese famine of 1959–61 in early life and long-term health conditions: A systematic review and meta-analysis. Int. J. Epidemiol..

[33] Song S. (2013). Identifying the intergenerational effects of the 1959–1961 Chinese Great Leap Forward Famine on infant mortality. Econ. Hum. Biol..

[34] Li Y., Sunder N. (2021). What doesn’t kill her, will make her depressed. Econ. Hum. Biol..

[35] Cortés-Albornoz M.C., García-Guáqueta D.P., Velez-van-Meerbeke A., Talero-Gutiérrez C. (2021). Maternal Nutrition and Neurodevelopment: A Scoping Review. Nutrients.

[36] Gray J.P., Müller V.I., Eickhoff S.B., Fox P.T. (2020). Multimodal Abnormalities of Brain Structure and Function in Major Depressive Disorder: A Meta-Analysis of Neuroimaging Studies. Am. J. Psychiatry.

[37] Januar V., Saffery R., Ryan J. (2015). Epigenetics and depressive disorders: A review of current progress and future directions. Int. J. Epidemiol..

[38] Hu X.F., Liu G.G., Fan M. (2017). Long-Term Effects of Famine on Chronic Diseases: Evidence from China’s Great Leap Forward Famine. Health Econ..

[39] Lin J.Y., Yang D. (1995). Food Availability, Entitlement and the Chinese Famine of 1959–61. Econ J.

[40] Song S., Wang W., Hu P. (2009). Famine, death, and madness: Schizophrenia in early adulthood after prenatal exposure to the Chinese Great Leap Forward Famine. Soc. Sci. Med..

[41] Mu R., Zhang X. (2011). Why does the Great Chinese Famine affect the male and female survivors differently? Mortality selection versus son preference. Econ. Hum. Biol..

[42] Ross C.E., Masters R.K., Hummer R.A. (2012). Education and the gender gaps in health and mortality. Demography.

[43] Chlapecka A., Kagstrom A., Cermakova P. (2020). Educational attainment inequalities in depressive symptoms in more than 100,000 individuals in Europe. Eur. Psychiatry J. Assoc. Eur. Psychiatr..

[44] Mizuno R. (2000). The male/female ratio of fetal deaths and births in Japan. Lancet.

