# Supplementary material for: A Link between Prenatal Stage of Life during the Great Chinese Famine and Subsequent Depressive Symptoms among Middle-Aged and Older Adults
Source: Nutrients. 2023 Oct 29;15(21):4600. doi: 10.3390/nu15214600 (PMC10647632; doi:10.3390/nu15214600)

Table S1. Characteristics of participants, CHARLS, 2011, *N* (%)

Characteristics	All participants (n=5391)			Pre-famine (n=1624)			Famine (n=1812)			Post-famine (n=1955)		
	Total	Rural (n=4782)	Urban (n=609)	Total	Rural (n=1456)	Urban (n=168)	Total	Rural (n=1588)	Urban (n=224)	Total	Rural (n=1738)	Urban (n=217)
Total												
Marital status												
Living with spouse	4758 (88.3)	4222 (88.3)	536 (88.0)	1432 (88.2)	1289 (88.5)	143 (85.1)	1595 (88.0)	1401 (88.2)	194 (86.6)	1731 (88.5)	1532 (88.2)	199 (91.7)
Living without spouse	633 (11.7)	560 (11.7)	73 (12.0)	192 (11.8)	167 (11.5)	25 (14.9)	217 (12.0)	187 (11.8)	30 (13.4)	224 (11.5)	206 (11.8)	18 (8.3)
Education												
Primary school and below	2639 (49.0)	2551 (53.4)	88 (14.4)	937 (57.7)	899 (61.7)	38 (22.6)	806 (44.5)	772 (48.6)	34 (15.2)	896 (45.8)	880 (50.6)	16 (7.4)
Junior high school and above	2752 (51.0)	2231 (46.6)	521 (85.5)	687 (42.3)	557 (38.3)	130 (77.4)	1006 (55.5)	816 (51.4)	190 (83.8)	1059 (54.2)	858 (49.4)	201 (92.5)
Female (n=2927)												
Marital status												
Living with spouse	2520 (86.1)	2229 (86.1)	291 (86.1)	706 (85.8)	631 (86.3)	75 (81.5)	850 (85.3)	753 (85.5)	97 (83.6)	964 (87.1)	845 (86.5)	119 (91.5)
Living without spouse	407 (13.9)	360 (13.9)	47 (13.9)	117 (14.2)	100 (13.7)	17 (18.5)	147 (14.7)	128 (14.5)	19 (16.4)	143 (12.9)	132 (13.5)	11 (8.5)
Education												
Primary school and below	1756 (60.0)	1703 (65.8)	53 (15.7)	589(71.6)	565 (77.3)	24 (26.1)	561 (56.3)	543 (61.6)	19 (15.5)	606 (54.7)	595 (60.9)	11 (8.5)
Junior high school and above	1171	886 (34.2)	285 (84.3)	687 (28.4)	166	68 (73.9)	436	338	98 (84.5)	501	382 (39.1)	119

	(40.0)				(22.7)		(43.7)	(38.4)		(45.3)		(91.5)
Male (n=2464)												
Marital status												
Living with spouse	2238 (90.8)	1993 (90.9)	245 (90.4)	726 (90.6)	658 (90.8)	68 (89.5)	745 (91.4)	648 (91.6)	97 (89.8)	767 (90.4)	687 (90.3)	80 (92.0)
Living without spouse	226 (9.2)	200 (9.1)	26 (9.6)	75 (9.4)	67 (9.2)	8 (10.5)	70 (8.6)	59 (8.4)	11 (10.2)	81 (9.6)	74 (9.7)	7 (8.0)
Education												
Primary school and below	883(35.8)	848 (36.7)	35 (12.9)	348 (43.4)	334 (46.1)	14 (18.4)	245 (30.1)	229 (32.4)	16 (14.8)	290 (34.2)	285 (37.4)	5 (5.8)
Junior high school and above	1581 (64.2)	1345 (61.3)	236 (87.1)	453 (56.6)	391 (53.9)	62 (81.6)	570 (69.9)	478 (67.6)	92 (85.2)	558 (65.8)	476 (62.5)	82 (94.2)

Table S2. Stratified distributions of CES-D 10 scores among participants, CHARLS, 2011, median (IQR)

Characteristics	Pre-famine (n=1624)			Famine (n=1812)			Post-famine (n=1955)		
	Total	Rural (n=1456)	Urban (n=168)	Total	Rural (n=1588)	Urban (n=224)	Total	Rural (n=1738)	Urban (n=217)
Female (n=2927)	8.0 (10.0)	8.0 (10.0)	4.0 (6.0)	7.0 (8.0)	8.0(9.0)	5.0 (5.0)	7.0 (8.0)	7.0 (8.0)	5.0 (6.0)
Marital status									
Living with spouse	7.0 (9.0)	8.0 (9.0)	4.0 (6.0)	7.0 (8.0)	7.0 (8.0)	5.0 (6.0)	7.0 (8.0)	7.0 (7.5)	4.0 (6.0)
Living without spouse	10.0 (11.0)	10.0 (11.0)	5.0 (11.0)	9.0 (10.0)	10.0 (9.0)	5.5 (4.0)	8.0 (9.0)	9.0 (9.0)	5.0 (6.0)
Education									
Primary school and below	9.0 (10.0)	9.0 (10.0)	6.0 (14.0)	8.0 (9.0)	8.0 (9.0)	6.0 (4.0)	8.0 (8.0)	8.0 (8.0)	7.0 (7.0)
Junior high school and above	5.0 (8.0)	6.0 (8.0)	4.0 (5.0)	6.0 (8.0)	7.0 (12.0)	5.0 (4.5)	6.0 (6.0)	6.0 (7.0)	4.0 (6.0)
Male (n=2464)	6.0 (7.0)	6.0 (8.0)	6.0 (8.0)	5.0 (8.0)	5.0 (7.0)	3.5 (6.0)	5.0 (7.0)	5.0 (8.0)	4.0 (4.5)
Marital status									
Living with spouse	5.0 (7.0)	5.0 (7.0)	5.5 (8.0)	5.0 (7.0)	5.0 (8.0)	3.0 (6.0)	5.0 (7.0)	5.0 (7.0)	3.0 (4.0)
Living without spouse	9.5 (10.5)	9.0 (11.5)	11.0 (4.5)	7.0 (9.0)	8.0 (9.0)	6.0 (13.0)	7.5 (11.0)	7.5 (11.0)	9.0 (10.0)
Education									
Primary school and below	6.0 (8.0)	6.0 (8.0)	6.0 (10.0)	7.0 (8.0)	7.0 (8.0)	3.0 (3.0)	7.0 (8.0)	7.0 (8.0)	7.5 (8.0)
Junior high school and above	5.0 (6.0)	5.0 (6.0)	6.0 (6.5)	4.0 (7.0)	5.0 (7.0)	4.0 (6.0)	4.0 (6.0)	4.0 (6.0)	4.0 (4.0)

Note: IQR, interquartile range.

Table S3. The placebo test of difference-in-differences estimates of prenatal famine exposure and depressive symptoms

	**Unadjusted β(95% CI)**	***p***	**Adjusted β(95% CI)**	***p***
Overall participants				
Control cohort (1949–1953)	Reference		Reference	
Placebo-treated cohort (1954–1958)	-0.28 (-3.23,2.68)	0.853	0.37 (-2.54,3.27) [a]	0.800
Rural				
Control cohort (1949–1953)	Reference		Reference	
Placebo-treated cohort (1954–1958)	0.22 (-3.09,3.54)	0.985	-0.86 (-7.55,5.83) [b]	0.775
Urban				
Control cohort (1949–1953)	Reference		Reference	
Placebo-treated cohort (1954–1958)	0.48 (-2.79,3.74)	0.801	0.79 (-5.78,7.37) [b]	0.813

Note: CI: confidence interval
[a] Adjusted for residence, sex, marital status, education;
[b] Adjusted for sex, marital status, education.

Figure S1. Flow chart of study participants

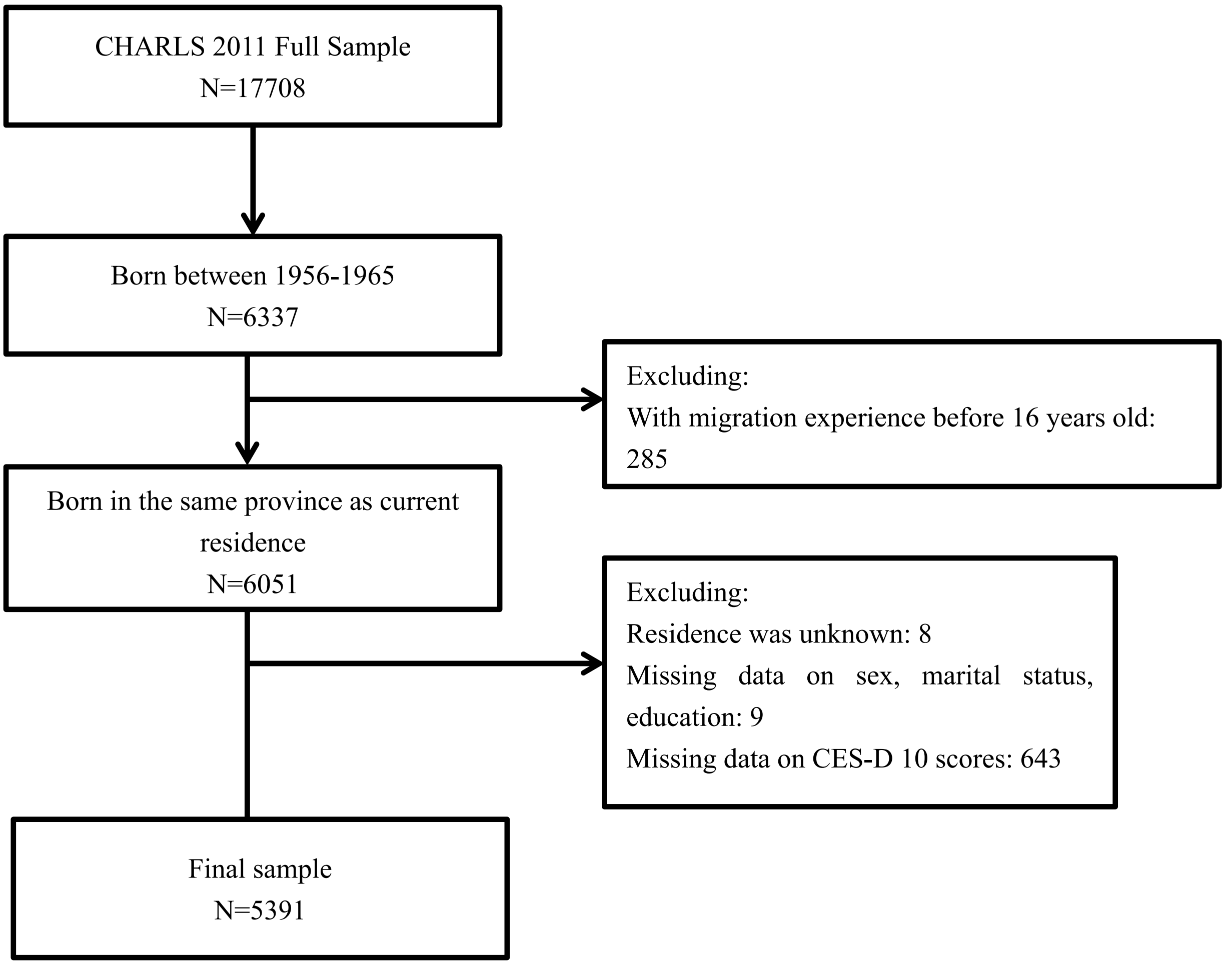

Supplement: Supplementary file 1 [file nutrients-15-04600-s001.zip › nutrients-2667336-supplementary.pdf]
